# Photoperiod-Treatment in Mediterranean Bucks Can Improve the Reproductive Performance of the Male Effect Depending on the Extent of Their Seasonality

**DOI:** 10.3390/ani11020400

**Published:** 2021-02-05

**Authors:** Luis A. Zarazaga, María Carolina Gatica, Manuel Delgado-Pertíñez, Horacio Hernández, José Luis Guzmán, José Alberto Delgadillo

**Affiliations:** 1 Departamento de Ciencias Agroforestales, Campus Universitario de la Rábida, Escuela Técnica Superior de Ingeniería, Universidad de Huelva, Carretera de Huelva-Palos de la Frontera, 21819 Huelva, Spain; guzman@uhu.es; 2 Facultad de Recursos Naturales Renovables, Universidad Arturo Prat, Avenida Arturo Prat, Iquique 2120, Chile; mgatica@unap.cl; 3 Departamento de Ciencias Agroforestales, Escuela Técnica Superior de Ingeniería Agronómica, Universidad de Sevilla, Ctra. Utrera km 1, 41013 Sevilla, Spain; pertinez@us.es; 4 Centro de Investigación en Reproducción Caprina, Universidad Autónoma Agraria Antonio Narro, Periférico Raúl López Sánchez y Carretera a Santa Fe, Torreón 27054, Mexico; hernandezhoracio@outlook.com (H.H.); joaldesa@yahoo.com (J.A.D.)

**Keywords:** male effect, Murciano–Granadina breed, Blanca Andaluza breed, seasonality, photoperiod, reproduction, fertility

## Abstract

**Simple Summary:**

On the extensive and semi-extensive goat farms of the Mediterranean region, the induction of the male effect is a common reproductive management practice in spring mating (seasonal anoestrous). The sexual activity displayed by the bucks is one of the main factors that determines the reproductive performance of this practice and, for that reason, it is essential to photostimulate the bucks prior to using it. However, the effectiveness of this photostimulation and the male effect could depend on the seasonality of the breed of buck used. Thus, the present work aimed to compare the efficiency of the male effect, in terms of doe reproductive response and reproductive performance, as induced by bucks made sexually active via photostimulation, from breeds with different reproductive seasonality (Murciano–Granadina, low reproductive seasonality vs. Blanca Andaluza, high reproductive seasonality). The results demonstrated that the Blanca Andaluza bucks subjected to a natural photoperiod and used for the male effect induced a lower percentage of females into oestrus and ovulation, resulting in lower productivity. This suggests that photoperiod-treated bucks efficiently induce the male effect, but photostimulation may be more necessary for breeds with deep seasonality.

**Abstract:**

This study examines the effectiveness of the photoperiod treatment (extra light for 88 days) to render the bucks sexually active during the seasonal anoestrous in order to induce a male effect, depending on the reproductive seasonality of the breed of the bucks used. In April, 57 anoestrous Blanca Andaluza does were distributed into four groups with three males each: 13 were exposed to control Murciano–Granadina bucks (lower seasonality); 15 were exposed to photostimulated Murciano–Granadina bucks; 14 were exposed to control Blanca Andaluza bucks (higher seasonality), and 15 were exposed to photostimulated Blanca Andaluza bucks. After male introduction, the sexual behaviour of the bucks was assessed, and harness marks recorded doe oestrous behaviour. Ovulation was confirmed from plasma progesterone, and the ovulation rate was assessed by transrectal ultrasonography. Fecundity, fertility, prolificacy and productivity were also determined. All of the does in all of the groups showed ovulation. Interaction between both sources of variation was observed: the percentage of females showing oestrous (*p* < 0.01) and productivity (*p* < 0.05) was the lowest in the Blanca Andaluza control group (50% and 0.36 ± 0.17 goat kids born/female, respectively). In conclusion, photoperiod-treated bucks efficiently induce a male effect, but photostimulation could be more necessary for breeds with deep seasonality.

## 1. Introduction

Most breeds of goat from temperate latitudes show seasonal variations in their sexual activity [1,2,3]. This is regulated by an endogenous rhythm, synchronised mainly by the photoperiod. In males, the short days and decreasing day length stimulate the secretion of the luteinising hormone (LH), which in turn, induces testicular growth and the release of testosterone, resulting in quantitative and qualitative improvements in semen production plus increased sexual behaviour. In contrast, long days and increasing day length reduce LH secretion and testicular growth, leading to a fall in the plasma testosterone concentration, reduced sperm quality, and diminished sexual behaviour [4,5,6,7,8,9,10].

In female goats, reproductive seasonality can be modified by sociosexual interactions with males. The introduction of males to seasonally anovulatory does, previously isolated from these males, can induce them to ovulate [11]. This is known as the “male effect”, and it has been extensively studied in sheep and goats; Delgadillo et al. [12] wrote an interesting review explaining this practice. Nevertheless, it has been demonstrated that the isolation between males and females is not necessary when sexually active males are used [13]. On the extensive and semi-extensive goat farms of the Mediterranean region, the induction of the male effect is a common reproductive management practice in spring mating. However, the degree of sexual behaviour displayed by the bucks determines the proportion of does that eventually ovulate [14,15,16]. To overcome this problem, exogenous melatonin [13,14,15,16] or photoperiod treatment could be used to induce the sexual activity of the bucks during spring by extra illumination for 2.5–3 months during the winter and followed by the natural photoperiod [17,18,19,20,21,22,23].

In Spain, there are a large number of native goat breeds. The Murciano–Granadina goat breed, which is well adapted to the Mediterranean environmental conditions, is the most important Spanish dairy goat breed, with a total of some 500,000 animals kept in the Andalusia regions (especially in Granada and Murcia). The Blanca Andaluza goat, an endangered Spanish meat goat breed, is also well adapted to the Mediterranean area. The bucks of Murciano–Granadina experience longer reaction times during the spring and variations on testosterone concentrations throughout the year [24,25,26] but these are lower than those of other Spanish breeds such as the Payoya [10,26] or the Blanca Andaluza breed [27]. The bucks of Blanca Andaluza showed an extended period of reproductive seasonality with basal testosterone concentrations from December to July [27] similar to those of the Payoya bucks [10].

We hypothesised that the effectiveness of the photoperiod treatment to induce sexual activity on bucks during the seasonal anoestrous depends on the seasonality of the breed of the bucks. The present work aimed to compare the efficiency, in terms of doe reproductive response and reproductive performance, of the male effect as induced by bucks from breeds with different reproductive seasonality, made sexually active via photostimulation.

## 2. Materials and Methods

### 2.1. Study Conditions

All animals were handled in pens with an open area and an enclosed zone. All methods were performed via prepared human resources in exact agreement with the Spanish rules for the insurance of investigational animals (RD 53/2013), and in concurrence with European Union Directive 86/609. The techniques of the current trial were assessed by the Certified Association of the Ethical Committee for Animal Experimentation (CEEA-OH) from the University of Granada and approved with the reference number 297-CEEA-OH-2018 and authorised by the Andalusia Regional Government with the number 22/05/2019/094. The study was conducted at the experimental farm of the University of Huelva (latitude 37°20′ N and longitude 6°54′ W), which meets the requirements of the European Community Commission for Scientific Procedure Establishments (2010/63). All animals were fed daily with lucerne hay, barley straw (ad libitum) and commercial concentrate to maintain their weight in agreement with INRA requirements [28]. All animals had free access to water and mineral supplement.

### 2.2. The Kind of Bucks Used to Induce the Male Effect: Low or High Reproductive Seasonality

We used four groups (n = 4 each) of sexually experienced Murciano–Granadina (MG) (low reproductive seasonality, from February to May) and Blanca Andaluza (BL) (high reproductive seasonality, from December to July) bucks to induce the male effect. All bucks were two years old at the beginning of the study. On the 13th of November, a group of males of each breed, held in open barns, were exposed to artificially long days (16-h day: 8-h night; lights on 0600; lights off 2200) for 88 days (photoperiod-treated bucks). The photoperiod was regulated by an electric timer that controlled white fluorescent strip lights providing approximately 200 lux at the level of a buck’s eye. At the end of the photoperiod treatment (i.e., on the 9th of February of the following year), the bucks were left under natural photoperiod conditions. The remainder of the bucks were exposed to the natural photoperiod during the whole experiment (control bucks). Bucks of each group were housed in separate pens and completely isolated from the does.

### 2.3. Preparation of Does

We used four groups of Blanca Andaluza females to induce the male effect (described previously). Fifty-seven, seasonally anovulatory goats, which were 2–3 years old at the beginning of the study were used. These females had previously delivered between September and October. Does were assigned randomly to one of four groups as follows: (1) does exposed to the Murciano–Granadina control bucks (MG Control; n = 13); (2) does exposed to the Murciano–Granadina photoperiod-treated bucks (MG Photo; n = 15); (3) does exposed to the Blanca Andaluza control bucks (BL Control; n = 14); 4) does exposed to the Blanca Andaluza photoperiod-treated bucks (BL Photo; n = 15). The females were kept together until the introduction of the males when the four groups were established. At this moment, they were housed in open barns completely isolated from the other groups.

### 2.4. The Male Effect

**Figure 1:**
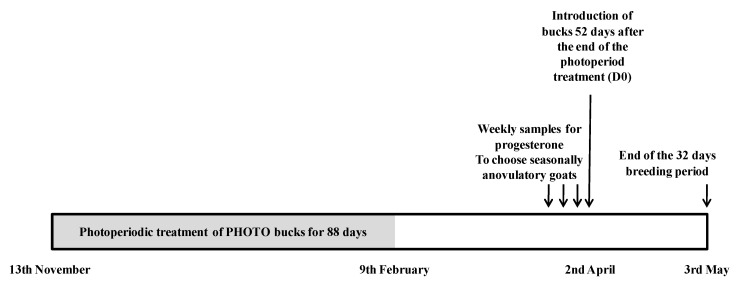
Experimental design of the male effect using bucks of Murciano–Granadina breed or Blanca Andaluza breed subjected to the photoperiodic treatment, i.e., long days for three months between November and February or not.

On the 2nd of April, 52 days after the end of the photoperiod treatment (as described in Section 2.2), three of the four males from each group of males were selected. During the previous week, the photostimulated bucks were exposed to does in oestrus (not the experimental does) for 5 min on one day, and their sexual behaviour was assessed by observing genital sniffing, nudging and mounting attempts. Three bucks which showed similar sexual behaviour, within the groups of photoperiod-treated bucks were selected and assigned to groups of does (MG Photo and BL Photo). Three bucks of each breed were chosen at random from the groups of untreated bucks and assigned to the control groups of does (MG Control and BL Control). After equipping them with marking harnesses, the bucks were placed in contact with the experimental does and kept with them for the following 32 days (until the 3rd of May) (Figure 1).

### 2.5. Variables Recorded for the Does

#### 2.5.1. The Detection of Oestrous Behaviour, Ovulation and Ovulation Rate

To monitor the ovulatory cyclicity of the females before their introduction to the males [Day 0 (D0); the 2nd of April], blood samples were collected once per week over three consecutive weeks, and the plasma progesterone concentrations were determined. Females with plasma progesterone concentrations ≤1.0 ng/mL in all samples before D0 were considered to be in anoestrous. Does with plasma progesterone concentrations ≥1.0 ng/mL in at least two consecutive samples were deemed to have ovulated and to have produced a corpus luteum of normal duration [1] and those females were discarded from the study.

Oestrous behaviour was recorded every day by direct visual observation of the marks left by the marking harnesses [29]. The interval between male introduction and first oestrous behaviour was calculated for each female.

After the introduction of the bucks, plasma progesterone concentration was determined twice per week to monitor the ovulatory response after the male introduction. The date of onset of a normal ovulatory response was defined as that of the first sample with progesterone concentrations above the baseline (≥1.0 ng/mL). Silent ovulation was deemed to have occurred when an increase in plasma progesterone above baseline was seen in at least one sample but was not preceded by oestrous behaviour. The percentages of females showing oestrus with or without ovulation, as well as those showing silent ovulation, were inferred from the plasma progesterone concentrations.

In all cases, blood was collected by jugular venepuncture in tubes containing 10 µL heparin; They were immediately centrifuged at 2300× *g* for 30 min at 4 °C, and the resultant plasma was stored at −20 °C until analysis.

The occurrence of ovulation and the ovulation rate were assessed by the number of corpora lutea observed in each female by transrectal ultrasonography conducted 6–8 days after the detection of oestrus [30]. The procedure was performed using an Aloka SSD-500 (Ecotron, Madrid, Spain) apparatus connected to a 7.5-MHz linear probe.

#### 2.5.2. Plasma Samples and Hormone Analysis

Plasma progesterone was determined using an enzyme-linked immunoassay kit (Ridgeway Science Ltd., Gloucester, UK) following the manufacturer’s instructions [29,30,31]. The sensitivity of the assay was 0.2 ng/mL. Intra- and inter-assay coefficients of variation for sample pools of 0.5 and 1 ng/mL were 4.1%, 7.9%, and 6.0%, 7.9%, respectively.

#### 2.5.3. Fecundity, Fertility, Prolificacy and Productivity

Fecundity (percentage of pregnant does/does mounted by the males) was determined via transrectal ultrasonography on day 45 after oestrous was displayed. Fertility (percentage of goats kidding per doe serviced), prolificacy (the number of kids born per female kidding) and productivity (the number of kids born per female serviced) were also determined [32].

### 2.6. Buck Plasma Testosterone and Sexual Behaviour

Blood for the determination of plasma testosterone was obtained and managed as described previously. Blood samples were taken weekly before D0 and twice a week after D0 at 09:00 h from the onset of the experiment (the 13th of November until the 4th of May). Testosterone concentrations were determined using a commercial enzyme-linked immunoassay kit (Demeditec Diagnostics, Kiel-Wellsee, Germany). The sensitivity of the assay was 0.1 ng/mL. Intra- and inter-assay coefficients of variation for sample pools of 1.0 and 6.0 ng/mL were 6.3%, 4.4%, and 6.2%, 4.6%, respectively.

The sexual behaviour of the bucks was also observed for 30 min (always from 8:00 a.m. to 8:30 a.m. and in all groups) on Days 0 to 9 post-introduction. Genital sniffing (when the buck sniffed the anogenital area of the doe); licking (when the buck licked the flanks of the doe); nudging (when the buck hit the doe with their legs); sneezing sounds (sounds emitted by the bucks); mounting attempts (when the buck attempted to mount the doe without intromission) and mounting with intromission (when the bucks mounted the doe with intromission) were all recorded. The sexual behaviour of all bucks was monitored using a video recording system, thus avoiding human interaction with the animals.

### 2.7. Statistical Analyses

The values for testosterone concentrations were examined by ANOVA with time as a repeated measure and the breed and the experimental treatment of the males (breed and photoperiod) as the main factors. The Tukey test was used to detect differences between groups each week.

The variables expressed as percentages—does showing ovulation, those showing oestrus behaviour and ovulation, fecundity and fertility—were analysed using multinomial logistic regression. Ovulation rates and prolificacy were compared using the Mann-Whitney U test. Productivity and the dates of ovulation, and ovulation with oestrus behaviour were compared by ANOVA with the breed and the male treatment as a fixed effect. The Duncan test was used to detect differences between experimental groups.

The percentage of genital sniffs, licks, nudges, sneezing sounds, mounting attempts, and mounting with intromission were calculated for each group and were analysed using the Fisher–Freeman–Halton exact probability test for multiple group comparisons, and the Fisher exact probability test for two-group comparisons as required.

Data are expressed as the mean ± SEM, and differences were considered significant at *p* ≤ 0.05. However, if *p*-values were between >0.05 and ≤0.10, a tendency for differences was defined. All calculations were performed using IBM SPSS Statistics for Windows (version 25.0; IBM Corp., Armonk, NY, USA).

## 3. Results

### 3.1. Testosterone Concentrations and Sexual Interactions with Bucks

**Figure 2:**
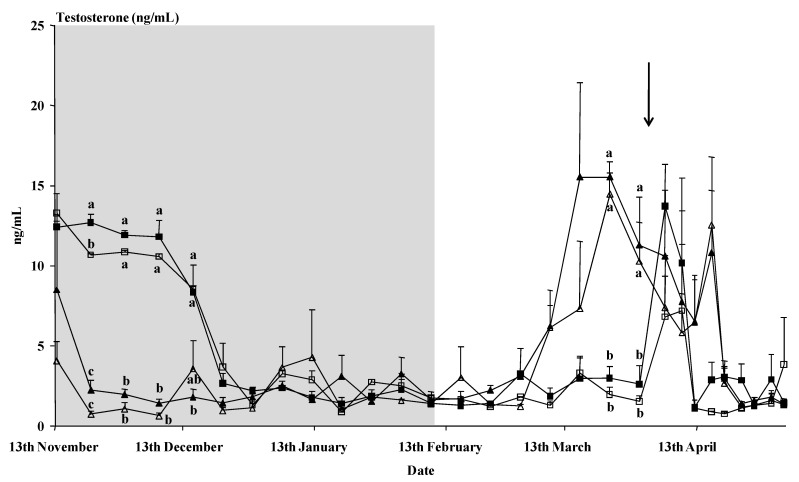
Plasma testosterone concentration (ng/mL) of bucks of Murciano–Granadina breed (filled symbols) or Blanca Andaluza breed (empty symbols) subjected to the photoperiodic treatment, i.e., long days for three months between November and February (photo; triangles) or not (control; squares). The shaded area indicates the time of the photoperiod treatment. The arrow indicates D0, when the male effect was applied. Values with different letters (a, b, c) are different (*p* < 0.05).

Time had a clear effect on the plasma testosterone concentration (*p* < 0.01), as did the interaction time x buck treatment (*p* < 0.01) (Figure 2). The photoperiod-treated bucks showed a rapid decrease in testosterone concentrations after the onset of the photoperiodic treatment. Those bucks showed higher testosterone concentrations than the control bucks from the 9th of March until the 16th of April, except for the samples of the 5th and 9th of April, just after the male introduction. No interaction time x breed or interaction time x buck treatment x breed was observed (*p* > 0.05). Moreover, on the whole, no effect of the buck treatment or the breed or an interaction buck treatment x breed was observed on the mean concentrations of testosterone (*p* > 0.05).

**Figure 3:**
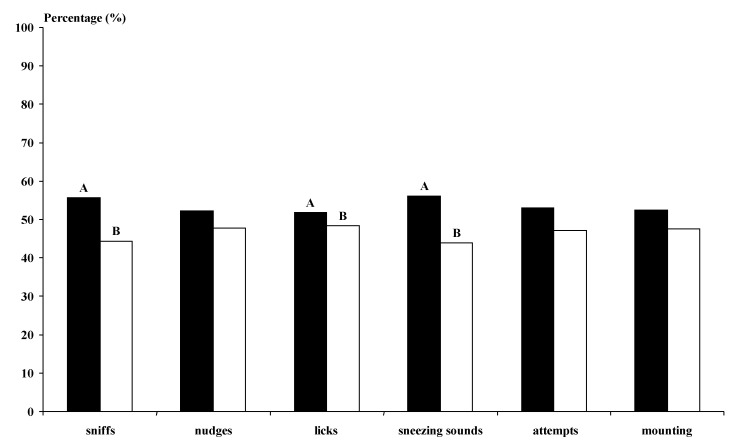
Types of sexual advance (%) performed by the males after females were submitted to the male effect using photoperiod-treated males (■, n = 6) or males exposed to the natural photoperiod (□, n = 6). Half of each group was composed of Murciano-Granadna bucks, and the other half was composed of Blanca Andaluza bucks.Different letters (A, B) differ significantly (*p* < 0.01). Sniffs (when the buck sniffed the anogenital area of the doe). Licks (when the buck licked the flanks of the doe). Nudges (when the buck kicked the doe). Sneezing sounds (sounds emitted by the bucks). Mounting attempts (when the buck attempted to mount the doe without intromission). Mounting with intromission (when the bucks mounted the doe with intromission).

**Figure 4:**
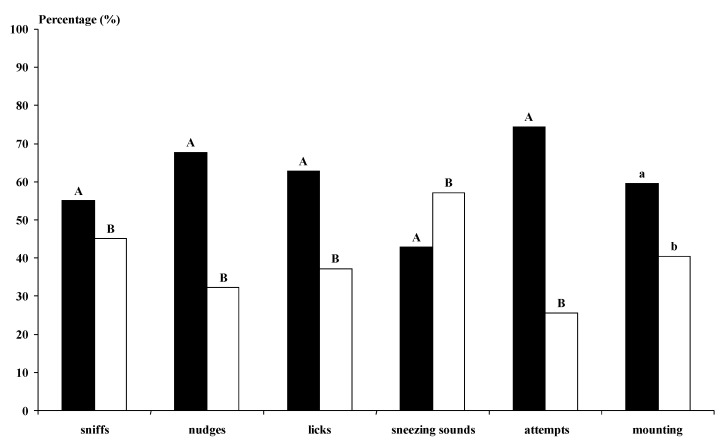
Types of sexual advance (%) performed by the males when females were submitted to the male effect using Murciano–Granadina (■, n = 6) or Blanca Andaluza bucks (□, n = 6). Half of each breed group was composed of photoperiod-treated males, and the other half was composed of males subjected to a natural photoperiod. Different letters differ significantly: a, b: (*p* < 0.05); A, B: (*p* < 0.01). Sniffs (when the buck sniffed the anogenital area of the doe). Licks (when the buck licked the flanks of the doe). Nudges (when the buck kicked the doe). Sneezing sounds (sounds emitted by the bucks). Mounting attempts (when the buck attempted to mount the doe without intromission). Mounting with intromission (when the bucks mounted the doe with intromission).

The photoperiod-treated bucks undertook more genital sniffs, licking and sneezing sounds than the not-treated bucks (*p* < 0.01) (Figure 3). The MG bucks undertook more behavioural interactions with the does than the BL bucks for all studied variables, except for the sneezing sounds (*p* < 0.01) (Figure 4). The MG Control and Photo bucks showed higher numbers of interactions with the does, except for sneezing sounds, than all other kinds of bucks and the BL Control bucks showed lower numbers of interactions than all other kinds of bucks (*p* < 0.05).

### 3.2. Doe Reproductive Response

All the females in all groups showed ovulation (Table 1), and the interval between male introduction and the first increase in the progesterone concentration was not modified by the kind of used male or by the interaction between breed x treatment of the males (*p* > 0.05).

For the percentage of females showing oestrous and ovulating, an interaction breed x treatment of the males was observed (92%, 87%, 50% and 100% for females in contact with MG Control, MG Photo, BL Control, BL Photo, respectively, *p* ≤ 0.01, Table 1) This interaction could be explained by the superior response of the group of females submitted to the male effect using Control bucks of the MG breed (92% vs. 50%, for MG and BL, respectively, *p* ≤ 0.01). In contrast, the response of the groups of females submitted to the male effect using photostimulated males was independent of the breed used (87% vs. 100%, for MG and BL, respectively, *p* ≥ 0.01). The main factors did not modify the interval introduction of males to first detected oestrous (*p* > 0.05, Table 1). The interval from male introduction to first oestrous behaviour was shorter than the interval from interval between male introduction and the first elevation of the progesterone concentration (6.43 ± 0.48 days vs. 10.63 ± 0.37 days; *p* < 0.01).

No interaction breed x treatment of the males for fertility was observed (*p* = 0.092). However, the percentage of fertility was significantly higher in the females submitted to the male effect using photostimulated bucks (80% vs. 52%, for Photo and Control groups, respectively, *p* < 0.05) and tended to be significant in the females submitted to the male effect using MG bucks (79% vs. 55%, for MG and BL, respectively, *p* = 0.052).

For productivity, an interaction was observed in breed x treatment of the males (1.08 ± 0.21, 1.00 ± 0.17, 0.36 ± 0.17, 1.13 ± 0.19 goat kids born by female present in the group for females in contact with MG Control, MG Photo BL Control, BL Photo, respectively, *p* ≤ 0.05; data not shown). This interaction could be explained because the productivity of the group of females submitted to the male effect using Control bucks was superior when the MG breed was used (1.08 ± 0.21 vs. 0.36 ± 0.17 goat kids born by female present in the group, for MG and BL, respectively, *p* ≤ 0.05). The groups of females submitted to the male effect using photostimulated males have no differences between them and were independent of the breed used (1.00 ± 0.17 vs. 1.13 ± 0.19 goat kids born by female present in the group, for MG and BL, respectively, *p* ≥ 0.05). Moreover, lower productivity was observed in the group of females submitted to the male effect using BL Control bucks.

None of the main studied factors modified the ovulation rate, fecundity, or prolificacy (Table 1).

## 4. Discussion

The results obtained in the present experiment confirm our hypothesis indicating that when the seasonality of the buck to induce the male effect is high, the sexual stimulation of their sexual activity using methods as photoperiodic treatments is more important. The results show that at Mediterranean latitudes the breed of the bucks used to induce the male effect and the treatment to which those males have been subjected (photostimulated with artificial long days for three months between November and February vs. a natural photoperiod) are important factors that change the percentage of females showing oestrous and productivity as a response to the male effect as a consequence of the interaction observed between both sources of variation. On the other reproductive variables studied (females showing ovulation, fecundity and fertility), no interaction between the sources of variation was observed and, as a consequence, they have independent effects on them.

The first remarkable and surprising result was the very high percentage of females showing elevation of progesterone concentrations after male introduction, because the 100% of the females responded, independent of the breed of the buck or the photoperiodic treatment received by the bucks. However, Delgadillo et al. [23] and Flores et al. [18] did not report any elevation of progesterone after teasing using bucks who were exposed to a natural photoperiod. Different and non-exclusive explanations could be suggested. First, we used a very high ratio of male:female, because we introduced 3 males per group of ~15 females. However, in a recent experiment using photostimulated males, we observed a similar ovulation response in groups with between 1:5 to 1:20 male:female ratios [19], which suggests the importance of the ratio male:female may be higher when the males are not photostimulated. In this way, we observed a lower percentage of females showing ovulation when 3 males were used in groups of 24–29 females [19]. Second, the introduction of the males perhaps induced a reactivation of the hypothalamus–pituitary–gonadal axis that increased the progesterone concentrations, but this reactivation did not induce an adequate oestrous response. In this study alone, we observed a reduction in the percentage of females showing oestrous in comparison to the percentage of females ovulating.

No differences were observed in the reproductive performances between the breeds of bucks used to induce the male effect, except for a tendency to be significant in the fertility percentage (*p* = 0.052). We used these breeds because the results of a literature review suggested that their breeding activity during the seasonal anoestrous was very different. The Murciano–Granadina breed is a Spanish breed that experiences a reduction in their reproductive activity from February to May [24], and the bucks experience longer reaction times during spring [25]. The Blanca Andaluza breed experiences seasonal anoestrous between the second fortnight of January to the second fortnight of August [3,27]. The bucks of this breed showed lower testosterone concentrations during winter and spring and also experienced longer reaction times during these seasons [27]. As described, the bucks and does of both breeds experience a reduction in their reproductive activity during seasonal anoestrous but the reduction is higher in the Blanca Andaluza breed. However, this reduction in the breeding activity did not induce any statistical differences between breeds in the present experiment, except for a tendency to be significant on the fertility of the does in contact with the Murciano–Granadina bucks. The lower seasonality of this breed could have induced this slightly higher fertility in the groups of does in contact with MG bucks. However, the treatment with photostimulation could have masked the possible differences between breeds because, at least in the variables where interaction between sources of variation was observed (females in oestrus, ovulation and productivity), the BL Control females showed lower results and higher values were observed in the BL Photo group. However, no statistical differences were observed with the groups of females bred with MG bucks. However, when we compared the results on the variables mentioned previously, only the results for the groups of females bred with control males were modified by the breed used and were lower in the BL Control group than in the MG Control group.

Similarly to other results published in the previous literature [18,23] and obtained by our group, the photostimulation of the males using long days results in a higher percentage of females showing oestrous, higher fertility and productivity (+37 kids per 100 goats serviced). This confirms the results suggesting that the reproductive condition of the buck is a key factor to induce a high reproductive response to the male effect in does. However, a very interesting result is the interaction observed between breed x treatment of the males for the percentage of females showing oestrous and productivity. In fact, in both reproductive performances, the MG Control group showed performances much higher than the BL Control group, but similar to the groups of females where photostimulated bucks were used to induce the male effect (independently of the breed). Thus, these results suggest that the photoperiodic treatment could be more efficient when used on breeds with a deep seasonal anoestrous. However, this treatment may not be strictly necessary when the breed of males used shows a reduced seasonality. In this way, in sheep, we have observed in a commercial farm using Merino breed, a breed with reduced seasonality, treated or not with photoperiod, that the fertility and productivity did not vary [33].

The testosterone concentrations cannot be used to explain the difference between the results of the same kind of male group and different breed, because no differences on the testosterone concentrations were observed. One possible explanation for this improvement of the reproductive performances could be the higher interactions between the males and the females observed in the group of females breeding with males of the MG Control group, in comparison to the females breeding with the BL Control males. The reason for a higher interaction with the females could be that the libido of the MG bucks was higher than the BL bucks.

Finally, the treatment with artificially long days for three months between November and February increases the testosterone concentration during what would normally be the sexually inactive period for goats at Mediterranean latitudes. Bucks treated in either way interacted more often with does than did no-treatment bucks, leading to greater doe reproductive performance and increasing the profitability in comparison to the use of males with natural sexual activity. This fact has been largely demonstrated by Delgadillo et al. in Mexico since 2002 [23], and this model could be used to induce the male effect on farms on which hormone treatments are prohibited.

According to the obtained results in the present study, the stimulation of the sexual activity of the bucks to induce a male effect under Mediterranean latitudes using artificial photoperiod is a necessary practice when the used breed shows a high extent of their seasonal anoestrous. This is feasible for goat producers in Southern Spain due to the availability of sufficient hours of sunshine and the possibility of installing solar panels on farms that represent a strong initial investment but reduce the cost of long-term treatment. Further research is needed to determine the costs/benefits of the treatment and the possibility of improving the reproductive results depending on the breed of female used.

## 5. Conclusions

At Mediterranean latitudes, the buck reproductive condition is an important factor determining the quality of response of does to the male effect. When the breed of buck shows a deep seasonality, as does the Blanca Andaluza breed, photostimulation is necessary. The treatment of Blanca Andaluza bucks with photoperiod allows a higher productivity (+77 kids per 100 goats serviced) than when the bucks are under natural photoperiod. However, when the breed of buck shows a low reproductive seasonality, as does the Murciano-Granadina breed, this treatment might be not necessary.

## Figures and Tables

**Table 1:** Reproductive response of does exposed to Murciano–Granadina (MG) or Blanca Andaluza (BL) bucks submitted to an artificial photoperiod to stimulate their reproductive activity (Photo) or a natural photoperiod (Control).

Variable	MG Control (N = 13)	MG Photo (N = 15)	BL Control (N = 14)	BL Photo (N = 15)	Breed	Treatment	Interaction
Females ovulating (%)	100	100	100	100	NS	NS	NS
Interval introduction of male-normal ovulation (days)	10.3 ± 0.8	11.7 ± 0.6	10.6 ± 0.8	9.9 ± 0.8	NS	NS	NS
Females in oestrus and ovulating (%)	92 a	87 a	50 b	100 a	NS	*	**
Interval introduction of male-oestrus (days)	4.5 ± 0.9	6.7 ± 0.5	7.2 ± 0.8	7.1 ± 1.2	NS	NS	NS
Ovulation rate (corpora lutea)	1.42 ± 0.19	1.62 ± 0.14	1.31 ± 0.13	1.21 ± 0.11	NS	NS	NS
Fecundity (%)	92	92	100	87	NS	NS	NS
Fertility (%)	77 a	80 a	29 b	80 a	0.052	*	NS
Prolificacy (kids born by female kidding)	1.40 ± 0.16	1.25 ± 0.13	1.17 ± 0.17	1.42 ± 0.15	NS	NS	NS
Productivity (kids by number of females in the group)	1.08 ± 0.21 a	1.00 ± 0.17 a	0.36 ± 0.17 b	1.13 ± 0.19 a	NS	NS	*

Statistical probability for comparisons: NS, not significant (*p* > 0.05); *, *p* ≤ 0.05; **, *p* ≤ 0.01. a, b: Different letters in the same row within each variable reflect significant differences at *p* < 0.05.

## Data Availability

The data presented in this study are available on request from the corresponding author.
